# Supplementary figures and images for: Implementation of a colorectal cancer screening intervention in Malaysia (CRC-SIM) in the context of a pandemic: study protocol
Source: BMJ Open. 2022 Sep 1;12(9):e058420. doi: 10.1136/bmjopen-2021-058420 (PMC9438210; doi:10.1136/bmjopen-2021-058420)

# Implementation Research Logic Model (IRLM) Project Title:

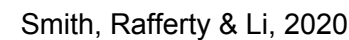

Supplement: Supplementary data [file bmjopen-2021-058420supp001.pdf]
